# Potentially Inappropriately Prescribed Medications Among Medicare Medication Therapy Management Eligible Patients with Chronic Kidney Disease: an Observational Analysis

**DOI:** 10.1007/s11606-020-06537-z

**Published:** 2021-01-27

**Authors:** Armando Silva-Almodóvar, Edward Hackim, Hailey Wolk, Milap C. Nahata

**Affiliations:** 1 Institute of Therapeutic Innovations and Outcomes (ITIO), The Ohio State University College of Pharmacy, Columbus, OH USA; 2 SinfoníaRx: A TRHC Solution, Tucson, AZ USA; 3 Department of Pediatrics, The Ohio State University College of Medicine, Columbus, OH USA

**Keywords:** Medicare, chronic kidney disease, medication therapy management, older adult

## Abstract

**Background:**

Potentially inappropriately prescribed medications (PIPMs) among patients with chronic kidney disease (CKD) may vary among clinical settings. Rates of PIPM are unknown among Medicare-enrolled Medication Therapy Management (MTM) eligible patients.

**Objectives:**

Determine prevalence of PIPM among patients with CKD and evaluate characteristics of patients and providers associated with PIPM.

**Design:**

An observational cross-sectional investigation of a Medicare insurance plan for the year 2018.

**Patients:**

Medicare-enrolled MTM eligible patients with stage 3–5 CKD.

**Main Measures:**

PIPM was identified utilizing a tertiary database. Logistic regression assessed relationship between patient characteristics and PIPM.

**Key Results:**

Investigation included 3624 CKD patients: 2856 (79%), 548 (15%), and 220 (6%) patients with stage 3, 4, and 5 CKD, respectively. Among patients with stage 3, stage 4, and stage 5 CKD, 618, 430, and 151 were with at least one PIPM, respectively. Logistic regression revealed patients with stage 4 or 5 CKD had 7–14 times the odds of having a PIPM in comparison to patients with stage 3 disease (*p* < 0.001). Regression also found PIPM was associated with increasing number of years qualified for MTM (odds ratio (OR) 1.46–1.74, *p* ≤ 0.005), female gender (OR 1.25, *p* = 0.008), and increasing polypharmacy (OR 1.30–1.57, *p* ≤ 0.01). Approximately 14% of all medications (2879/21093) were considered PIPM. Majority of PIPMs (62%) were prescribed by physician primary care providers (PCPs). Medications with the greatest percentage of PIPM were spironolactone, canagliflozin, sitagliptin, levetiracetam, alendronate, pregabalin, pravastatin, fenofibrate, metformin, gabapentin, famotidine, celecoxib, naproxen, meloxicam, rosuvastatin, diclofenac, and ibuprofen.

**Conclusion:**

Over one-third of Medicare MTM eligible patients with CKD presented with at least one PIPM. Worsening renal function, length of MTM eligibility, female gender, and polypharmacy were associated with having PIPM. Majority of PIPMs were prescribed by PCPs. Clinical decision support tools may be considered to potentially reduce PIPM among Medicare MTM–enrolled patients with CKD.

**Supplementary Information:**

The online version contains supplementary material available at 10.1007/s11606-020-06537-z.

## INTRODUCTION

Approximately 20% of adults above 60 years of age have chronic kidney disease (CKD).^1^ In 2016, Medicare spent roughly $12 billion in prescription drug coverage on patients with CKD, representing 20% of Medicare Part D drug spending.^1^ Patients with CKD represented a portion of the Medicare population at greater risk for hospitalization^1^, hospital readmission,^1^ polypharmacy, and consequently medication-related problems.^2,3^ Reducing prevalence of potentially inappropriately prescribed medications (PIPMs) may reduce avoidable morbidity and health care utilization among patients with CKD.

One registry-based study found 43% of older adults with stage 3 CKD and 58% of patients with stage 4 CKD required a medication dose adjustment based on renal function.^4^ In this same study, 9% of patients with stage 3 CKD and 38% of patients with stage 4 CKD required a medication discontinuation.^4^ Hanlon et al.^5^ reported 6–12% of older adults in a nursing home had PIPM based on renal function. A systematic review by Dorks et al.^6^ found prevalence of PIPM ranged from 1 to 37% in outpatient settings. Finally, another study identified PIPM use among 50–70% of adults with CKD in the United States (US).^7^ The high prevalence of PIPM among older adults with CKD suggests the need for dedicated clinical decision support tools (CDST) to assist prescribers in identifying PIPM. The use of CDST may be helpful given only 35% of patients in one study had CKD as a listed medical problem despite having an estimated glomerular filtration rate (eGFR) below 60 ml/min per 1.73 m^2^.^8^ Kurani et al.^7^ found 40–90% of patients were unaware of their CKD.

Presently, there are no studies describing rates of PIPM utilizing large databases of a Medicare-enrolled population. Additionally, there is no evidence about which providers may benefit from CDST to reduce rates of PIPM. The objectives of this investigation were to (1) determine the prevalence of PIPM among Medicare-enrolled patients with CKD participating in a Medication Therapy Management (MTM) program; (2) evaluate associations between patient characteristics with PIPM; and (3) identify providers that were most likely to prescribe PIPMs.

## METHODS

### Study Design

This was a retrospective cross-sectional analysis of a MTM provider’s database (SinfoníaRx). Analysis was limited to patients from one Medicare plan provider. Patients without laboratory data reflecting serum creatinine were excluded. Renal function (eGFR) was calculated with the chronic kidney disease-epidemiology collaboration CKD-EPI equation.^9^ CKD-EPI equation was utilized in contrast to the modification of diet in renal disease (MDRD) equation given its greater accuracy.^10^ EGFR was used to determine the patient’s stage of chronic kidney disease.^11^ Given renal damage could not be assessed, patients with stage 1 or 2 CKD were excluded. For this study, GFR and creatinine clearance were used interchangeably based on recommendations by the National Kidney Foundation for renal adjustment of medications.^12^ This study was approved by The Ohio State University Institutional Review Board as a retrospective record review.

### Data Source

Patient data from January 1 to December 31, 2018, in SinfoníaRx’s database were obtained. Information acquired included demographic information (age, gender, and zip code), prescription claims with medication names, doses, routes of administration, prescriber national provider identifier (NPI), duration of therapy, and laboratory data, specifically serum creatinine. Investigators were provided with the years a patient was qualified for MTM, and the number of unique prescribers and medications in the four months prior to MTM qualification. The prescriber NPI was cross-referenced with the base provider enrollment file published by the Centers for Medicare and Medicaid Services (CMS) to determine specialty.^13^ Zip code was used to determine the percentage of individuals who lived below the federal poverty line (FPL) within a patient’s zip code utilizing data published by the Census Bureau.^14^

### Study Population

This study evaluated patients who were Medicare-enrolled and MTM eligible. MTM eligibility was determined on a rolling basis throughout the year through assessment of certain chronic conditions, prescription claims for a specific number of medications, and annual medication cost exceeding a predetermined threshold.^15^ Patients enrolled in a MTM program may receive an annual review of their medications and receive quarterly reviews for specific medications. Medications targeted for specific review are determined by the patient’s insurance plan. It is important to note all patients may not have qualified on the same date. A 4-month assessment period of medications was used to determine if a Medicare-enrolled patient would qualify for MTM.

### Medications Assessed

Prescription claims covered by the insurance plan in the 4 months prior to MTM qualification were assessed in this investigation. Medications assessed for being potentially inappropriately prescribed medications (PIPMs) were those deemed relevant to the management of chronic diseases prevalent among older adults.^16,17^ These included diabetes, hypertension, heart failure, fluid retention, osteoporosis, anxiety, depression, arrhythmia, bipolar disorder, schizophrenia, hyperlipidemia, urinary incontinence, acid reflux, seizure, neuropathy, arthritis, psoriatic arthritis, rheumatoid arthritis, pain, allergies, atrial fibrillation, Parkinson’s disease, Alzheimer’s disease, and gout. Only systemic medications were included in the analysis.

Appendix Table 1 lists the medications and renal dosing guidelines for the present investigation. To ensure the findings of the present study to be as relevant to clinical practice as possible, medications were considered PIPM when Lexicomp dosing information stated a medication “should be avoided,” “use is contraindicated,” “use is not recommended,” and “consider alternative,” or when a dose is adjusted to a “max dose” based on a patient’s GFR or creatinine clearance. Lexicomp database was used given it is a tertiary reference that is continuously updated.^18^ Patients with an eGFR below 15 ml/min per 1.73 m^2^ were assumed to be on dialysis. Medications for these patients were considered PIPM if the dose exceeded dosing recommendations for patients on hemodialysis or peritoneal dialysis, the dose exceeded dosing recommendations for patients with higher eGFR and the medications were considered non-dialyzable or unlikely to be dialyzed, or the medication was contraindicated for a higher GFR.^18,19^ Medications noted as non-dialyzable or unlikely to be dialyzed are included in Appendix Table 1. Medications were also used to calculate a patient’s chronic disease burden using the Rx-Risk Comorbidity Index.^20^

### Statistical Analysis

Data were coded and organized using Microsoft Excel (2016 MSO, Redmond, WA) and IBM SPSS software (v26.0, IBM Corp, Armonk, NY). Medians with interquartile ranges and counts with percentages were used as appropriate to describe the population. Counts and percentages were used to describe medications assessed and that were identified as PIPM, and the prevalence of PIPM by prescriber specialty. In this analysis, age (under 65, 65–74, and greater than 75 years), stage of CKD (stage 3, 4, and 5), percent of individuals below the FPL (0.00–9.99%, 10–19.99%, 20–29.99%, 30–39.99%, and 40–100%), years qualified to receive MTM (1, 2, 3, and 4), number of medications (8–10, 11–13, 14–16, and ≥ 17), number of prescribers (1–3, 4–6, 7–9, and ≥ 10), and chronic disease burden (2–4, 5–7, 8–10, and ≥ 11) were transformed into ordinal variables.

**Figure 1:**
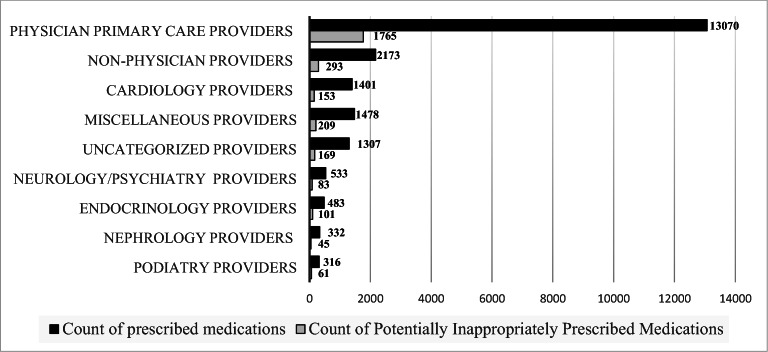
Count of prescription claims that require renal adjustment and count of potentially inappropriately prescribed medications (PIPMs) by provider specialty. Specialty individually included if with more than 40 PIPMs.

**Figure 2:**
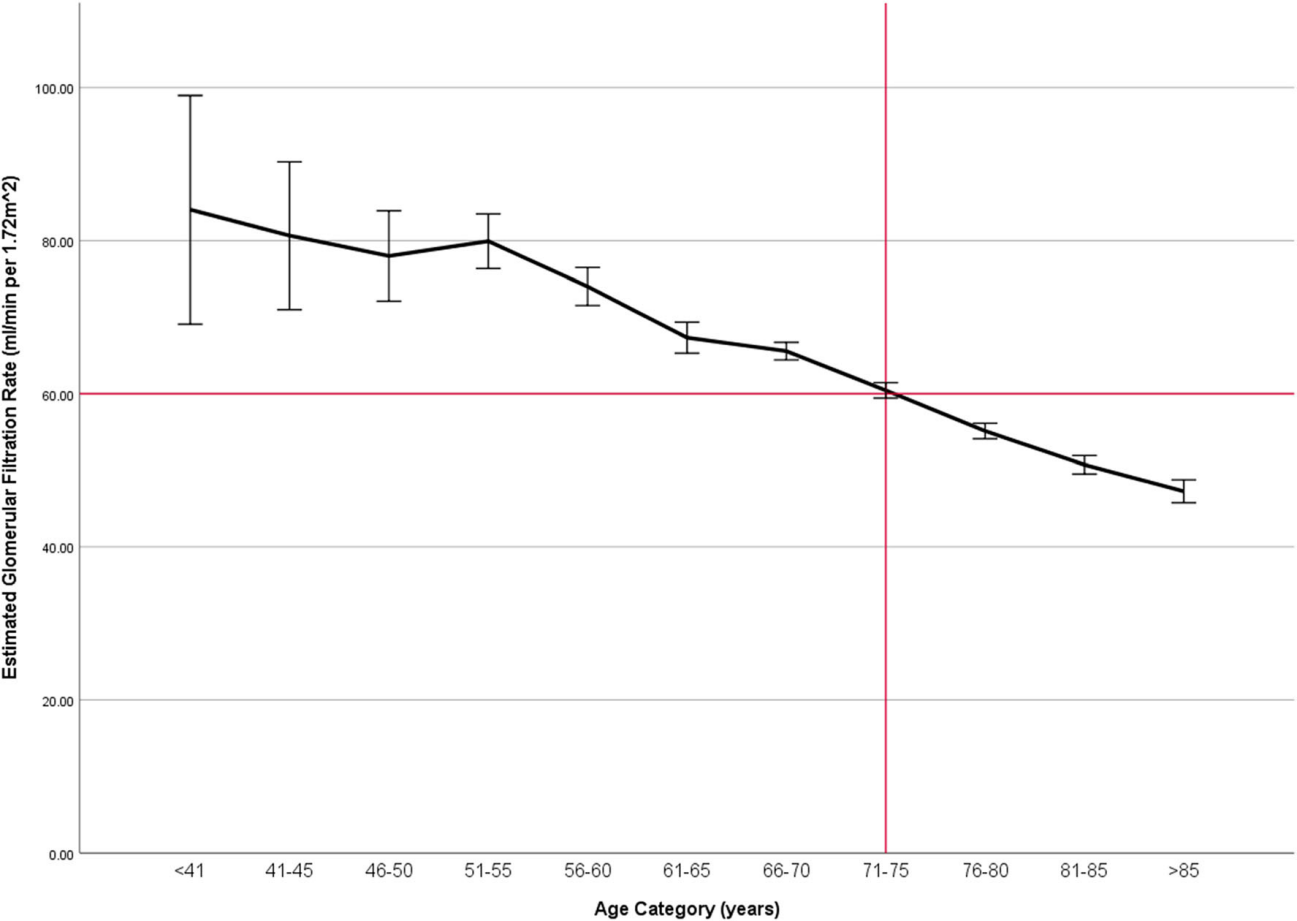
Illustration of the relationship between age and average glomerular filtration rate (GFR) by age category. Error bars represent the 95% confidence interval. Red line denotes when a patient may be diagnosed with stage 3 chronic kidney disease.

Chi-square or Fisher exact tests were used to assess differences between patients with PIPM versus patients that did not. A logistic regression determined the relationship between patient characteristics and prescription of at least one PIPM. The regression included age, sex, number of medications, number of prescribers, percentage of individuals under the FPL, stage of CKD, chronic disease burden, and years qualified to receive MTM. Post hoc exploratory testing via chi-square and odds ratios evaluated the relationship between number of prescribers and disease burden with CKD stage. Also assessed was the relationship between disease burden with number of prescribers and length of MTM eligibility. A two-tailed a priori value of 0.05 was used. Figures illustrated count of renally adjusted medications and PIPM by specialty (Fig. 1) and the relationship between age and GFR (Fig. 2).

## RESULTS

The original sample included 7442 adults. Of these, 3800 (51%) patients with a GFR greater than 60 ml/min per 1.73 m^2^ and 18 (0%) patients without a serum creatinine value were excluded. The final analysis included 3624 (49%) patients. This cohort was predominantly female (2129, 59%); 57% were with 8 or more chronic diseases, with a median age of 76 years (interquartile range (IQR) 71–82 years), had a median of 6 (IQR 4–9) prescribers, and were prescribed a median of 11 (IQR 9–14) medications. Complete demographic information can be found in Table 1.

**Table 1:** Demographic Data of Patients with Stage 3–5 Chronic Kidney Disease Between Those With and Without a Potentially Inappropriately Dosed Medication (PIPM)

Characteristics	Without a PIPM, *n* = 2425 (67%)	With a PIPM, *n* = 1199 (33%)	*p* value	Total population, *N* = 3624
	*n* (%)	*n* (%)		*N* (%)
Stage of chronic kidney disease				
3	2238(92)	618(51)	< 0.001	2856(79)
4	118(5)	430(36)		548(15)
5	69(3)	151(13)		220(6)
Age (years)				
Under 65	183(8)	117(10)	0.03	300(8)
65–74	772(32)	400(33)		1172(32)
> 75	1470(61)	682(57)		2152(59)
Sex				
Female	1398(58)	731(61)	0.06	2129(59)
Male	1027(42)	468(39)		1495(41)
Percent of individuals below the federal poverty level (FPL)				
0.00–9.99	242(10)	114(10)	0.99	356(10)
10–19.99	906(37)	449(38)		1355(37)
20–29.99	795(33)	397(33)		1192(33)
30–39.99	407(17)	202(17)		609(17)
40–100	67(3)	35(3)		102(3)
Years qualified to receive Medication Therapy Management services				
1	708(29)	207(17)	< 0.001	915(25)
2	399(17)	193(16)		592(16)
3	401(17)	230(19)		631(17)
4	917(38)	569(48)		
Number of medications				11(9–14)
8–10	1158(48)	402(34)	< 0.001	1560(43)
11–13	661(27)	349(29)		1010(28)
14–16	327(14)	229(19)		556(15)
≥ 17	279(12)	219(18)		498(14)
Number of unique prescribers				
1–3	491(20)	190(16)	< 0.001	681(19)
4–6	945(39)	395(33)		1340(37)
7–9	596(25)	315(26)		911(25)
≥ 10	393(16)	299(25)		692(19)
Rx-Risk Comorbidity Index (chronic disease burden)				
2–4	145(6)	45(4)	< 0.001	193(5)
5–7	1015(42)	362(30)		1337(38)
8–10	967(40)	500(42)		1467(41)
≥ 11	298(12)	289(24)		587(16)

There were 2856 (79%), 548 (15%), and 220 (6%) patients with stage 3, 4, and 5 CKD, respectively. Among these patients, 1199 (33%) were prescribed at least one PIPM. There were 618, 430, and 151 patients with stage 3, 4, and 5 CKD respectively, prescribed at least one PIPM. Patients with stage 4 and 5 CKD had 11 times the odds of having been prescribed at least one PIPM compared to patients with stage 3 CKD (odds ratio (OR) 11.25, 95% confidence interval (CI) 9.33–13.57, *p* < 0.001).

Significant differences identified via univariate testing between patients with and without a PIPM included stage of CKD (*p* < 0.001), age (*p* = 0.03), years qualified to receive MTM (*p* < 0.001), number of medications (*p* < 0.001), and number of unique prescribers (*p* < 0.001) (see Table 1).

Logistic regression found female sex, years qualified for MTM, number of medications, and stage of CKD remained significant (*p* ≤ 0.008) as presented in Table 2. Females had 1.25 times the odds of having PIPM when compared to males (OR 1.25 (95% CI 1.06–1.48), *p* = 0.008). Patients eligible for MTM services for more than a year compared to recently eligible patients had 1.46 to 1.74 times the odds of having PIPM (*p* ≤ 0.005). Patients with 11 or more medications when compared to patients with fewer medications had 1.30–1.57 times the odds of having PIPM (*p* ≤ 0.01). Patients with stage 4 and 5 CKD were with 7.44–13.54 times the odds of having PIPM when compared to patients with stage 3 CKD (*p* < 0.001). Further results are presented in Table 2.

**Table 2:** Results from Logistic Regression Assessing the Relationship Between Unique Patient Characteristics with Having at Least One Potentially Inappropriately Dosed Medication (*N* = 3624)

Characteristic	Odds ratio (95% confidence interval)	*p* value
Age		
< 65 (reference)	-	-
65–74	0.91(0.67–1.23)	0.53
> 75	0.76(0.57–1.02)	0.07
Sex		
Male (reference)	-	0.008
Female	1.25(1.06–1.48)	
Poverty quintile (%)		
0.00–9.99 (reference)	-	-
10.00–19.99	1.07(0.80–1.43)	0.64
20.00–29.99	1.12(0.83–1.50)	0.46
30.00–39.99	0.98(0.71–1.36)	0.90
40.00–100	1.42(0.84–2.39)	0.19
Years qualified to receive Medication Therapy Management services		
1 (reference)	-	-
2	1.46(1.12–1.90)	0.005
3	1.74(1.34–2.25)	< 0.001
4	1.52(1.22–1.90)	< 0.001
Number of medications		
8–10 (reference)	-	-
11–13	1.30(1.05–1.60)	0.01
14–16	1.57(1.21–2.03)	0.001
≥ 17	1.54(1.14–2.06)	0.005
Number of prescribers		
1-3 (reference)	-	-
4–6	0.82(0.65–1.04)	0.10
7–9	0.82(0.63–1.06)	0.12
≥ 10	1.05(0.80–1.40)	0.71
Stage of chronic kidney disease		
3 (reference)	-	-
4	13.54(10.76–17.04)	< 0.001
5	7.44(5.46–10.14)	< 0.001
Rx-Risk Comorbidity Index (chronic disease burden)		
2–4 (reference)	-	-
5–7	0.99(0.66–1.48)	0.96
8–10	1.06(0.70–1.60)	0.79
≥ 11	1.56(0.98–2.49)	0.06

A total of 11,4333 medications were assessed. Among these medications 21,093 were identified as potentially requiring renal adjustment, with 2879 (14%) identified as PIPM. Seventeen medications accounted for at least 10% of prescriptions considered PIPM and with at least 100 overall prescription claims: spironolactone (153/397, 39%), canagliflozin (49/142, 35%), sitagliptin (591/1743, 34%), levetiracetam (39/135, 29%), alendronate (99/431, 23%), pregabalin (75/352, 21%), pravastatin (62/298, 21%), fenofibrate (68/329, 21%), metformin (469/2428, 19%), gabapentin (348/1945, 18%), famotidine (93/562, 17%), celecoxib (24/175, 14%), naproxen (58/481, 12%), meloxicam (69/574, 12%), rosuvastatin (147/1228, 12%), diclofenac (29/259, 11%), and ibuprofen (71/624, 11%). Complete information is found in Appendix Table 1.

Appendix Table 2 and Figure 1 describe by specialty the count of medications that may need renal adjustment and PIPM. Physician primary care providers (PCPs) prescribed the greatest number of prescriptions potentially requiring renal adjustment (13070/21093, 62%) and PIPM (1760/2854, 61%), while the greatest percentage of PIPM by specialty occurred among endocrinologists (101/483, 21%).

Figure 2 demonstrated the average GFR falling below 60 ml/min per 1.72 m^2^ between 71 and 75 years of age. Patients with stage 4 and 5 CKD presented with 1.65 times the odds of having more than 7 prescribers when compared to patients with stage 3 CKD (OR 1.65 (95% CI 1.41–1.94), *p* < 0.001). Additionally, patients with stage 4 and 5 CKD had 1.72 times the odds of having 8 or more chronic diseases when compared to patients with stage 3 CKD (OR 1.72, 95% CI 1.45–2.03). Patients with 8 or more chronic diseases had 4 times the odds of having more than 7 prescribers when compared to patients with fewer diseases (OR 4.16, 95% CI 3.60–4.80). Patients qualified for a CMR for 3 and 4 years were with 2.20 times the odds of having 8 or more chronic diseases when compared to individuals who were qualified for fewer years (OR 2.20, 95% CI 1.92–2.51).

## DISCUSSION

To our knowledge, this is the first study that utilized insurance prescription claims paired with laboratory data to identify PIPMs among Medicare-enrolled older adults with CKD. We found approximately one-third of older adults with stage 3–5 CKD had at least 1 PIPM. Patients with stage 4 and 5 CKD were with 11 times the odds of having a PIPM when compared to patients with stage 3 CKD. These findings highlighted the need for more frequent review of medications for patients as their kidney function declines. It is also important to highlight the medications used in this study were limited to those relevant to ambulatory care settings^16^ and older adults^17^, using clinically relevant dosing recommendations from a continuously updated and widely used tertiary source, Lexicomp.^18^

The prescribers with the greatest number of PIPM were PCPs, while endocrinologists presented with the highest percent of PIPM. Finally, positively associated with presence of PIPM were increasing polypharmacy, female gender, increasing years qualified for MTM, and worsening CKD. These findings highlighted which patients and prescribers may benefit from CDST dedicated to identifying PIPM.

MTM services implemented at the insurance plan level serve as a safety net for health care systems seeking to broadly apply medication management services to their populations. Despite multiple studies reporting the implementation of dedicated services within a health care setting to reduce prevalence of PIPM in patients with CKD,^2,8,21–29^ the findings from this study emphasized the need to implement services for monitoring outside of individual health care settings. This may be related in part to incomplete medical records in individual settings, given one study found only 35% of patients with a calculated eGFR less than 60 ml/min per 1.73 m^2^ had CKD as a medical problem in their health record.^8^ Additionally, inconsistent implementation of CDST may contribute to the ineffective identification and reduction of PIPMs in different settings.^27–29^ Thus, health care systems should partner with providers in outpatient settings to determine how to identify patients with CKD and improve the identification of PIPM.

As patients with worsening renal function were more likely to have more prescribers and greater disease burden, the monitoring of prescription claims is another means of overcoming limitations associated with a fragmented health care system. At the insurance prescriptions claims level, a provider may utilize software to receive a patient’s latest serum creatinine measurements to estimate their GFR. Subsequent data feeds of prescription claims can be monitored for medications highlighted as PIPM. Once a PIPM is identified, the prescriber can be notified through electronic communication. This would ensure prescribers have access to relevant clinical information for the care of their patients.

This solution may be especially helpful to older adults, given 44% of the patients had more than 7 prescribers. If relevant health care data is not fully integrated between different providers or health care systems, an individual provider’s ability to make the most informed health care–related decision can be impeded. For example, this study found endocrinologists to proportionally have the highest percent of PIPM. This may have occurred due to the prescribing of anti-hyperglycemic medications (canagliflozin, sitagliptin, and metformin) and anticonvulsants (gabapentin and pregabalin) relevant to the comprehensive care of patients with diabetes, which encompassed 5 of the top 10 medications that presented with the highest proportion of PIPM. Endocrinologists may not know a patient is with CKD if relevant data for this patient is in a different health care system.

It is important to highlight that, while specific Medicare-enrolled adults may be eligible for MTM, lenient goals set by CMS could result in some patients never benefitting from the service.^30^ This may partly explain why length of MTM eligibility was associated with presence of PIPM. Furthermore, in this study, patients who were MTM eligible for a greater length of time presented with greater disease burden.

This study found Medicare-enrolled MTM eligible patients above the mean age of 71 years were associated with a GFR beginning to drop below 60 ml/min per 1.72 m^2^. This important finding may signal patients over the age of 71 years may benefit from a review of their medicines based on renal function at least annually. This initiative, especially in primary care settings, may lead to the timely identification of PIPMs. The medications associated with the greatest percentage of PIPM were spironolactone, canagliflozin, sitagliptin, levetiracetam, alendronate, pregabalin, pravastatin, fenofibrate, metformin, gabapentin, famotidine, naproxen, celecoxib, meloxicam, rosuvastatin, diclofenac, and ibuprofen. These medications should be especially targeted among patients with CKD.

Consistent with a previous review,^6^ polypharmacy and female gender were associated with higher PIPM. This study did not find age to be associated with PIPM use. This may have been due to the relationship between age and GFR which was unaccounted for in previous studies.^6^ The logistic regression did not find a significant relationship between number of prescribers and disease burden with PIPM. This may have occurred because of the strength of the relationship between CKD stage with PIPM and the confounding relationship between CKD stage with disease burden and number of prescribers.

### Limitations

Findings from this study were limited to a Medicare-enrolled MTM eligible population from one Medicare insurance plan; thus, prevalence of PIPM may differ from that of the overall Medicare population. Given the retrospective nature of the study, investigators could not communicate with prescribers to determine if renal function had been taken into consideration, and it was not possible to assess medication adherence. Patients were assumed Caucasian for calculations given race and ethnicity data were unavailable. Prevalence of PIPM may change over time given dosing strategies may be updated due to new published data. This study utilized the Rx-Risk Comorbidity Index to measure disease burden. The ability of this index to measure disease burden may be limited as medications assessed were those covered by the patient’s insurance plan. Medications covered by insurance were made available through electronic prescription claims; thus, over the counter medications not covered by the insurance could not be assessed. This analysis could not determine if a patient had an acute kidney injury at the time their renal function was assessed.

## CONCLUSION

One-third of the Medicare-enrolled MTM eligible patients with CKD were prescribed at least one PIPM in this investigation. The monitoring of prescription claims at the insurance plan level is one means of identifying and resolving use of these medications. The presence of PIPM was associated with worsening CKD, female sex, increasing polypharmacy, and MTM eligibility for multiple years. Patients over the age of 71 years of age may benefit from a targeted review of medications to assess the presence of PIPM due to decline in renal function.

## Data Availability

The data that support the findings of this study are available from SinfoníaRx but restrictions apply to the availability of these data, and so are not publicly available. Data are however available from the authors upon reasonable request and with permission of SinfoníaRx.

## Supporting Information

ESM 1 (DOCX 48 kb)
