# Supplementary material for: Potentially Inappropriately Prescribed Medications Among Medicare Medication Therapy Management Eligible Patients with Chronic Kidney Disease: an Observational Analysis
Source: J Gen Intern Med. 2021 Jan 27;36(8):2346–52. doi: 10.1007/s11606-020-06537-z (PMC8342663; doi:10.1007/s11606-020-06537-z)
Supplement: ESM 1 — (DOCX 48 kb) [file 11606_2020_6537_MOESM1_ESM.docx]

Appendix Table 1: List of medications and dosing recommendations per changes in glomerular filtration rate or creatinine clearance.

| Disease State | Class | Medication | eGFR/CrCL | | Dose Adjustment | Count of PIPM | Total Count of Medications |
| --- | --- | --- | --- | --- | --- | --- | --- |
| diabetes | biguanide | metformin | eGFR | 30-45 | Max 1000 mg/day | 469 | 2428 |
|  |  |  |  | <30 | Use is contraindicated |  |  |
|  | sulfonylurea | glyburide^a^ | eGFR | <60 | Not recommended in CKD | 20 | 20 |
|  |  | glimepiride^a^ | eGFR | <15 | Consider alternative | 27 | 560 |
|  |  | glipizide (IR)^b^ | eGFR | <50 | Max 20 mg/day | 9 | 543 |
|  | dipeptidyl peptidase 4 inhibitor | alogliptin^a^ | CrCl | 30-60 | Max 12.5 mg/day | 13 | 13 |
|  |  |  |  | <30 | Max 6.25 mg/day |  |  |
|  |  |  |  | <15, hemodialysis | Ma 6.25mg/day |  |  |
|  |  | Saxagliptin | eGFR | <45 | Max 2.5 mg/day | 5 | 12 |
|  |  |  |  | <15, hemodialysis | Max 2.5mg/day |  |  |
|  |  | saxagliptin (Kombiglyze XR) | eGFR | 45-30 | Max 2.5 mg/day | 0 | 1 |
|  |  |  |  | <30 | Use is contraindicated |  |  |
|  |  | sitagliptin (Januvia)^a^ | eGFR | 30-45 | Max 50 mg/day | 476 | 1362 |
|  |  |  |  | <30 | Max 25 mg/day |  |  |
|  |  |  |  | Hemodialysis or peritoneal dialysis | Max 25mg/day |  |  |
|  |  | sitagliptin (Janumet)^a^ | eGFR | <45 | Max 50 mg/day | 106 | 317 |
|  |  |  |  | <30 | Use is contraindicated |  |  |
|  |  | sitagliptin (Janumet XR)^a^ | eGFR | 30-45 | Max 50 mg/day | 9 | 64 |
|  |  |  |  | <30 | Use is contraindicated |  |  |
|  | glucagon-like peptide-1 receptor agonist | exenatide IR (Byetta^)b^ | CrCl | <30 | Use is not recommended | 0 | 4 |
|  |  |  |  | <15 | Use is not recommended |  |  |
|  |  | exenatide ER (Bydureon) ^)b^ | eGFR | <45 | Use is not recommended | 28 | 62 |
|  |  |  |  | <15 | Use is not recommended |  |  |
|  |  | lixisenatide^)b^ | eGFR | <15 | Not recommended | 0 | 11 |
|  | sodium-glucose cotransporter 2 inhibitor | Dapagliflozin | eGFR | 15-30 | Max 5mg/day | 2 | 129 |
|  |  |  |  | <15, hemodialysis | Use is Contraindicated |  |  |
|  |  | canagliflozin^a^ | eGFR | 15-60 | Max 100 mg/day | 49 | 142 |
|  |  |  |  | <15, hemodialysis | Use is contraindicated |  |  |
|  |  | Empagliflozin | eGFR | <30 | Use is contraindicated | 4 | 36 |
|  |  |  |  | <15, hemodialysis | Use is contraindicated |  |  |
|  |  | Ertugliflozin | eGFR | <30 | Use is contraindicated | 0 | 0 |
|  |  |  |  | <15, hemodialysis | Use is contraindicated |  |  |
| hypertension | angiotensin-converting enzyme inhibitor | lisinopril | CrCl | <30 | Max 40 mg/day | 6 | 1364 |
|  |  |  |  | hemodialysis | Max 40mg/day |  |  |
|  |  | benazepril^a^ | CrCl | <30 | Max 40 mg/day | 3 | 198 |
|  |  |  |  | Hemodialysis, peritoneal dialysis | Max 20mg/day 50% usual dose |  |  |
|  |  | enalapril^)b^ | eGFR | <30 | Max 40mg/day | 2 | 503 |
|  | beta blocker | Atenolol | CrCl | 15-35 | Max 50 mg/day | 44 | 397 |
|  |  |  |  | <15, peritoneal dialysis | Max 25 mg/day |  |  |
|  |  |  |  | hemodialysis | Max36mg/day |  |  |
|  |  | betaxolol | eGFR | <30, hemodialysis | Max 20 mg/day | 0 | 0 |
|  |  | Nadolol | CrCl | >50 | Administer every 24 hours | 0 | 26 |
|  |  |  |  | 31-50 | Administer every 24-36 hour |  |  |
|  |  |  |  | 10-30 | Administer every 24-48 hours |  |  |
|  |  |  |  | <10, hemodialysis, peritoneal dialysis | Administer every 40-60 hours |  |  |
|  | diuretic, thiazide | hydrochlorothiazide^a^ | CrCl | <10 | Use is not recommended | 28 | 1947 |
|  |  | chlorthalidone^a^ | CrCl | <10 | Avoid use | 1 | 327 |
|  |  | indapamide^a^ | eGFR | ≤50 | Max 2.5 mg/day | 0 | 11 |
|  | mineralcorticoid receptor antagonist | spironolactone (tablet)^a^ | eGFR | 30-50 | Max 25mg/day | 153 | 397 |
|  |  |  |  | <30 | Not recommended |  |  |
|  |  | eplerenone^a^ | eGFR | 49-31 | Max 25mg/day | 8 | 26 |
|  |  |  |  | <30 | Use is contraindicated |  |  |
|  | potassium sparing diuretic | amiloride | CrCl | <30 | Use is contraindicated | 0 | 1 |
|  |  | Triamterene | CrCl | <30 | Use is contraindicated | 4 | 36 |
| arrhythmia | antiarrhythmic, class Ia | disopyramide IR^a^ | CrCl | 50-60 | Max 600 mg/day | 0 | 0 |
|  |  |  |  | 10-50 | Max 400 mg/day |  |  |
|  |  |  |  | <10 | Max 200 mg/day |  |  |
|  |  |  |  | <15 | Max 100 mg/day |  |  |
|  |  | disopyramide CR^a^ | CrCl | 41-60 | Max 400 mg/day | 0 | 0 |
|  |  |  |  | ≤40 | Use is not recommended |  |  |
|  | antiarrhythmic, class II | sotalol^b^ | CrCl | 30-60 | Max 320mg/day (administer every 24hours) | 0 | 21 |
|  |  |  |  | <30 | Max 160mg.day (administer every 48 hours) |  |  |
|  |  | acebutolol | CrCl | 25-49 | Max 600mg/day  (Reduce dose by 50%) | 0 | 0 |
|  |  |  |  | <25 | Max 300mg/day  (Reduce dose by 75%) |  |  |
|  | antiarrhythmic, class III | dofetilide | CrCl | 40-60 | Max 500mcg/day | 0 | 8 |
|  |  |  |  | 20-39 | Max 250mcg/day |  |  |
|  |  |  |  | <20 | Use is contraindicated |  |  |
| psychiatric | selective serotonin reuptake inhibitor | brexpiprazole^a^ | CrCl | <60 | Max 3mg/day | 0 | 5 |
|  |  | paroxetine (IR) ^a^ | CrCl | <30 | Max 40 mg/day | 0 | 109 |
|  |  | paroxetine (ER) ^a^ | CrCl | <30 | Max 50 mg/day | 0 | 6 |
|  | serotonin/norepinephrine reuptake inhibitor | desvenlafaxine^a^ | CrCl | 30-50 | Max 50 mg/day | 0 | 8 |
|  |  |  |  | <30 | Max 25 mg/day |  |  |
|  |  |  |  | <15, hemodialysis | Max 25 mg/day |  |  |
|  |  | duloxetine^a^ | CrCl | <30 | Max 60mg/day | 1 | 237 |
|  |  | levomilnacipran^a^ | CrCl | 30-59 | Max 80 mg/day | 0 | 2 |
|  |  |  |  | 15-29 | Max 40 mg/day |  |  |
|  |  |  |  | <15 | Use not recommended |  |  |
|  |  | milnacipran^a^ | CrCl | 15-30 | Max 100 mg/day | 0 | 0 |
|  |  |  |  | <15 | Use not recommended |  |  |
|  | dopamine/norepinephrine-reuptake inhibitor | bupropion XL^a^ | eGFR | <60 | Use of bupropion XL 450mg is not recommended | 0 | 0 |
|  | antimanic agent | lithium^b^ | CrCl | <30 | Avoid use | 0 | 17 |
|  | second generation (atypical) antipsychotic | paliperidone  (oral) ^b^ | CrCl | 50-60 | Max 6 mg/day | 0 | 0 |
|  |  |  |  | 10-49 | Max 3 mg/day |  |  |
|  |  |  |  | <10 | Use is not recommended |  |  |
|  |  | palperidone (IM) ^b^ | CrCl | <50 | Use is not recommended | 0 | 2 |
|  |  | cariprazine ^b^ | CrCl | <30 | Use not recommended | 0 | 0 |
|  |  | lurasidone^a^ | CrCl | <50 | Max 80mg/day | 0 | 14 |
| hyperlipidemia | HMG-CoA reductase inhibitor | rosuvastatin^a^ | CrCl | <30 | Max 10 mg/day | 147 | 1228 |
|  |  | pravastatin ^a^ | CrCl | <30 | Max 10 mg/day | 62 | 298 |
|  |  | pitavastatin^a^ | eGFR | <60 | Max 2 mg/day | 3 | 8 |
|  | fibric acid | gemfibrozil^a^ | eGFR | 10-50 | Max 900mg/day | 25 | 49 |
|  |  |  |  | <10 | Max 600mg/day |  |  |
|  |  | fenofibrate^a^ | CrCl | <30 | Use is contraindicated | 68 | 329 |
| osteoporosis | bisphosphonate | alendronate^a^ | CrCl | <35 | Use is not recommended | 99 | 431 |
|  |  | ibandronate^b^ | CrCl | <30 | Use is not recommended | 0 | 8 |
|  |  | risedronate^b^ | CrCl | <30 | Use is not recommended | 1 | 3 |
|  |  | zoledronic acid | CrCl | <30 | Use is not recommended (for non-oncology purposes) | 1 | 1 |
| genitourinary | anticholinergic agent | fesoterodine^b^ | CrCl | <30 | Max 4 mg/day | 0 | 7 |
|  |  | solifenacin^a^ | CrCl | <30 | Max 5 mg/day | 3 | 72 |
|  |  | tolterodine (IR)^a^ | CrCl | 10-30 | Max 2 mg/day (1 mg twice daily) | 0 | 10 |
|  |  | tolterodine ER^a^ | CrCl | 10-30 | Max 2 mg/day | 3 | 30 |
|  |  |  |  | <10 | Use is not recommended |  |  |
|  |  | trospium (IR)^b^ | CrCl | <30 | Max 20 mg/day | 2 | 17 |
|  |  | trospium ER^b^ | CrCl | <30 | Use is not recommended | 0 | 1 |
|  | beta-3 agonist | mirabegron^a^ | CrCl | 15-29 | Max 25 mg/day | 12 | 101 |
|  |  |  |  | <15 | Use is not recommended |  |  |
|  | alpha-1 antagonist | silodosin^a^ | CrCl | 30-50 | Max 4 mg/day | 7 | 16 |
|  |  |  |  | <30 | Use is contraindicated |  |  |
|  | phosphodiesterase-5 enzyme inhibitor | tadalafil (once-daily regimen)^a^ | CrCl | 30-50 | Max 5 mg/day | 1 | 12 |
|  |  |  |  | <30 | Use is not recommended |  |  |
|  |  |  |  | 15, hemodialysis | Use is not recommended |  |  |
| gastrointestinal | histamine H2 antagonist | cimetidine^a^ | eGFR | 10-50 | Max 800mg/day | 0 | 1 |
|  |  |  |  | <10 | Max 600mg/day |  |  |
|  |  | famotidine^a^ | CrCl | 30-60 | Max 40 mg/day | 93 | 562 |
|  |  |  |  | <30, hemodialysis, peritoneal dialysis | Max 20 mg/day |  |  |
|  |  | ranitidine^a^ | CrCl | <50,hemodialysis | Max 300 mg/day | 3 | 663 |
|  |  | nizatidine^a^ | CrCl | 10-50 | Max 150 mg/day | 0 | 0 |
|  |  |  |  | <10, hemodialysis, peritoneal dialysis | Max 75 mg/day  (150 mg every other day) |  |  |
| immunologic | phosphodiesterase-4 enzyme inhibitor | apremilast^b^ | CrCl | <30 | Max 30 mg/day | 1 | 5 |
| neuropathy | gamma-aminobutyric acid (GABA) Analog | gabapentin (IR) | CrCl | 50-60 | Max 1800mg/day | 348 | 1940 |
|  |  |  |  | 30-49 | Max 900 mg/day |  |  |
|  |  |  |  | 15-29 | Max 600 mg/day |  |  |
|  |  |  |  | <15 | Max 300 mg/day |  |  |
|  |  |  |  | hemodialysis | Max 900mg/week |  |  |
|  |  |  |  | peritoneal dialysis | Max 300mg/day |  |  |
|  |  |  |  | Continuous renal replacement therapy | Max 600mg/day |  |  |
|  |  | gabapentin ER (Gralise) | CrCl | 30-59 | Max 1800mg/day | 0 | 2 |
|  |  |  |  | <30, hemodialysis, peritoneal dialysis | Use is not recommended |  |  |
|  |  | gabapentin enacarbil (Horizant) | CrCl | 30-59 | Max 1200 mg/day | 0 | 3 |
|  |  |  |  | 15-30 | Max 600mg/day |  |  |
|  |  |  |  | <15 | Max 300mg/day |  |  |
|  |  |  |  | hemodialysis | Max 1800mg/week |  |  |
|  |  | pregabalin (IR) | Cr | 30-60 | Max 300 mg/day | 75 | 352 |
|  |  |  |  | 15-30 | Max 150 mg/day |  |  |
|  |  |  |  | <15 | Max 75 mg/day |  |  |
|  |  |  |  | hemodialysis | Max 75mg/day + 150mg after hemodialysis |  |  |
|  |  | pregabalin ER | CrCl | 30-60 | Max 330 mg/day | 0 | 0 |
|  |  |  |  | <30, hemodialysis | Use not recommended |  |  |
| pain | opioid analgesic | tramadol (IR)^a^ | CrCl | <30 hemodialysis, peritoneal dialysis | Max 200 mg/day | 9 | 482 |
|  |  | tramadol ER | CrCl | <30,hemodialysis, peritoneal dialysis | Avoid use | 0 | 5 |
|  |  | tapentadol^a^ | CrCl | <30 | Use not recommended | 0 | 0 |
|  |  | meperidine^a^ | CrCl | <60 | Avoid use | 0 | 0 |
| allergy | histamine H1 antagonist | acrivastine^b^ | CrCl | ≤48 | Avoid use | 0 | 0 |
|  |  | Cetirizine^a^(oral only) | eGFR | 31-11 | Max 5 mg/day | 0 | 2 |
|  |  |  | eGFR | <10 | Use is Contraindicated |  |  |
|  |  |  |  | hemodialysis | Max 5mg/day |  |  |
|  |  | levocetirizine^a^ | CrCl | 50-80 | Max 2.5 mg/day | 88 | 88 |
|  |  |  |  | 30-50 | Max 1.25 mg/day (2.5 mg every other day) |  |  |
|  |  |  |  | 10-30 | Max 0.83 mg/day (2.5 mg every 3-4 days) |  |  |
|  |  |  |  | <10. Hemodialysis, peritoneal dialysis | Use is contraindicated |  |  |
|  |  | loratadine | CrCl | 10-50 | Max 10mg/day | 0 | 0 |
|  |  |  |  | <10, hemodialysis | Max 5mg/day |  |  |
|  |  |  |  | Continuous renal replacement therapy | Max 10mg/day |  |  |
| insomnia | hypnotic | chloral hydrate | eGFR | <60 | Use is contraindicated | 0 | 0 |
|  |  | zopiclone^a^ | eGFR | <60 | Max 5mg/day at bedtime | 0 | 0 |
| atrial fibrillation | factor Xa inhibitor (oral) | dabigatran | CrCl | 15-30 | Max 150mg/day | 8 | 70 |
|  |  |  |  | <15, hemodialysis | Avoid use |  |  |
|  |  | edoxaban^a^ | CrCl | 15-50 | Max 30mg/day | 0 | 0 |
|  |  |  |  | <15, hemodialysis | Use is not recommended | 0 | 0 |
|  |  | rivaroxaban^a^ | CrCl | <15,hemodialysis, peritoneal dialysis | Avoid use | 13 | 374 |
| Parkinson’s disease | dopamine agonist | amantadine^a^ (IR) | CrCl | 30-50 | Max 100 mg/day | 19 | 30 |
|  |  |  |  | 15-29 | Max 50 mg/day  (100 mg every other day) |  |  |
|  |  |  |  | <15 | Max 200 mg/week |  |  |
|  |  |  |  | Hemodialysis, peritoneal dialysis | Max 200 mg/week |  |  |
|  |  |  |  | Continuous renal replacement therapy | 100mg/day |  |  |
|  |  | amantadine^a^ (ER capsule) | CrCl | 30-60 | Max dose 137 mg/day | 2 | 2 |
|  |  |  |  | 15-30 | Max 68.5 mg/day |  |  |
|  |  |  |  | <15 | Use is contraindicated |  |  |
|  |  |  |  | Hemodialysis | Use is contraindicated |  |  |
|  |  | amantadine^a^ (ER tablet) | CrCl | 30-50 | Max 161 mg/day  (322 mg every other day) | 0 | 0 |
|  |  |  |  | 15-30 | Max 80.5 mg/day  (322 mg every 96 hours) |  |  |
|  |  |  |  | <15 | Use is contraindicated |  |  |
|  |  |  |  | hemodialysis | Use is contraindicated |  |  |
|  |  | pramipexole^a^ (IR) | CrCl | 30-50 | Max 2.25 mg/day  (0.75 mg 3 times daily) | 1 | 35 |
|  |  |  |  | 15-29 | Max 1.5 mg/day |  |  |
|  |  |  |  | <15 | Has not been studied. |  |  |
|  |  | pramipexole^a^ (ER) | CrCl | 30-50 | Max 2.25 mg/day | 0 | 0 |
|  |  |  |  | <30 | Use not recommended |  |  |
|  |  |  |  | <15, hemodialysis | Use not recommended |  |  |
| arthritis | Nonsteroidal Anti-inflammatory Drug (NSAID) | celecoxib^a^ | eGFR | <30 | Use is not recommended | 24 | 175 |
|  |  | naproxen^a^ | eGFR | <30 | Avoid use | 58 | 481 |
|  |  | diclofenac^a^ | eGFR | <30 | Avoid use | 29 | 259 |
|  |  | ibuprofen^a^ | eGFR | <30 | Avoid use | 71 | 624 |
|  |  | diflunisal^a^ | eGFR | <30 | Avoid use | 0 | 0 |
|  |  | etodolac^a^ | eGFR | <30 | Avoid use | 1 | 10 |
|  |  | fenoprofen^a^ | eGFR | <30 | Avoid use | 1 | 1 |
|  |  | floctafenine | eGFR | <30 | Use is contraindicated | 0 | 0 |
|  |  | flurbiprofen^a^ | eGFR | <30 | Avoid use | 0 | 1 |
|  |  | indomethacin^a^ | eGFR | <30 | Avoid use | 4 | 7 |
|  |  | ketoprofen^a^ | eGFR | <30 | Avoid use | 0 | 2 |
|  |  | ketorolac^a^ (oral only) | eGFR | <30 | Avoid use | 0 | 1 |
|  |  | meclofenamate^a^ | eGFR | <30 | Avoid use | 0 | 0 |
|  |  | mefenamic^a^ acid | eGFR | <30 | Avoid use | 0 | 0 |
|  |  | meloxicam^a^ | eGFR | <30 | Avoid use | 69 | 574 |
|  |  | nabumetone^a^ | CrCl | 30-50 | Max 1500mg/day | 5 | 58 |
|  |  |  | eGFR | <30 | Avoid use |  |  |
|  |  | oxaprozin^a^ | eGFR | <30 | Avoid use | 0 | 0 |
|  |  | piroxicam^a^ | eGFR | <30 | Avoid use | 6 | 22 |
|  |  | sulindac^a^ | eGFR | <30 | Avoid use | 2 | 13 |
|  |  | tiaprofenic acid^a^ | eGFR | <30 | Avoid use | 0 | 0 |
|  |  | tolmetin^a^ | eGFR | <30 | Avoid use | 0 | 0 |
| epilepsy | anticonvulsant | levetiracetam (IR) | CrCl | 50-60 | Max 2000 mg/day | 39 | 135 |
|  |  |  |  | 30-50 | Max 1500 mg/day |  |  |
|  |  |  |  | 15-30 | Max 1000 mg/day |  |  |
|  |  |  |  | <15 | Max 500 mg/day |  |  |
|  |  |  |  | hemodialysis | Max 1000mg/day |  |  |
|  |  |  |  | peritoneal dialysis | Max 500 mg/day |  |  |
|  |  | levetiracetam ER | CrCl | 50-60 | Max 2000mg/day | 2 | 3 |
|  |  |  |  | 30-50 | Max 1500 mg/day |  |  |
|  |  |  |  | <30 | Max 1000 mg/day |  |  |
|  |  |  |  | hemodialysis | Use is not recommended |  |  |
|  |  |  |  | Peritoneal dialysis | Use is not recommended |  |  |
|  |  | zonisamide^b^ | GFR | <50 | Use is not recommended | 2 | 3 |
|  |  | ezogabine^b^ | CrCl | <50 | Max 600 mg/day | 0 | 0 |
|  |  | perampanel^a^ | CrCl | <30 | Use not recommended | 0 | 0 |
| gout | xanthine oxidase inhibitor | febuxostat^a^ | CrCl | <30 | Ma 40 mg/daily | 5 | 121 |
| Total |  |  |  |  |  | 2879 | 21093 |

Chronic Kidney Disease= CKD

Instant Release= IR

Extended Release= ER

Controlled Release=CR

ESRD: End Stage Renal Disease

Estimated Glomerular Filtration Rate= eGFR

Creatinine Clearance= CrCl

^A^Not dialyzable or unlikely to be dialyzable

^b^omitted from consideration for patients with an eGFR below 15ml/min per 1.73m^2^ due to being dialyzable or likely to be dialyzed

Appendix Table 2: Count of prescribed medications that may require renal adjustment and medications that were potentially inappropriately dosed (PIPM) by provider specialty among patients with Stage 3-5 Chronic Kidney Disease

| Specialty Category | Count of PIPM (N=2857) | Count of prescribed medications (N=20918) | Percentage of PIPM |
| --- | --- | --- | --- |
| **Physician Primary Care Providers** | **1765** | **13070** | **14%** |
| Family Medicine | 398 | 2787 | **14%** |
| General Practice | 20 | 133 | **15%** |
| Geriatric Medicine | 41 | 190 | **22%** |
| Internal Medicine | 1290 | 9870 | **13%** |
| Osteopathic Manipulative medicine | 1 | 4 | **25%** |
| Pediatric Medication | 15 | 86 | **17%** |
| **Non-physician Providers** | **293** | **2173** | **13%** |
| Nurse Practitioner | 167 | 1137 | **15%** |
| Physician Assistant | 125 | 1035 | **12%** |
| Other Non-physician | 1 | 1 | **100%** |
| **Cardiology Providers** | **153** | **1401** | **11%** |
| Advanced Heart Failure and Transplant Cardiology | 0 | 17 | **0%** |
| Cardiac Electrophysiology | 0 | 15 | **0%** |
| Cardiology | 152 | 1323 | **11%** |
| Interventional Cardiology | 1 | 46 | **2%** |
| **Endocrinology Providers** | **101** | **483** | **21%** |
| **Neurology/Psychiatry Providers** | **83** | **533** | **16%** |
| Neurology | 73 | 376 | **19%** |
| Psychiatry | 10 | 157 | **6%** |
| **Nephrology Providers** | **45** | **332** | **14%** |
| **Miscellaneous Providers** | **209** | **1478** | **14%** |
| Allergy Immunology | 5 | 11 | **45%** |
| Anesthesiology | 17 | 94 | **18%** |
| Cardiac Surgery | 0 | 1 | **0%** |
| Dentist | 4 | 34 | **12%** |
| Dermatology | 5 | 12 | **42%** |
| Diagnostic Radiology | 0 | 3 | **0%** |
| Emergency Medicine | 17 | 158 | **11%** |
| Gastroenterology | 17 | 110 | **15%** |
| General Surgery | 2 | 15 | **13%** |
| Hand Surgery | 0 | 4 | **0%** |
| Hematology/Oncology | 7 | 67 | **10%** |
| Hospice/Palliative Care | 1 | 2 | **50%** |
| Hospitalist | 3 | 35 | **9%** |
| Infectious Disease | 4 | 51 | **8%** |
| Intensivist | 1 | 2 | **50%** |
| Interventional Pain Management | 4 | 28 | **14%** |
| Interventional Radiology | 1 | 1 | **100%** |
| Maxillofacial Surgery | 0 | 2 | **0%** |
| Medical Oncology | 0 | 1 | **0%** |
| Neurosurgery | 3 | 20 | **15%** |
| Obstetrics/Gynecology | 5 | 25 | **20%** |
| Ophthalmology | 7 | 11 | **64%** |
| Oral Surgery | 5 | 36 | **14%** |
| Orthopedic Surgery | 13 | 118 | **11%** |
| Other-physician | 0 | 3 | **0%** |
| Otolaryngology | 1 | 25 | **4%** |
| Pain Management | 14 | 63 | **22%** |
| Pathology | 0 | 1 | **0%** |
| Physical Medicine and Rehabilitation | 14 | 123 | **11%** |
| Plastic and reconstructive surgery | 0 | 1 | **0%** |
| Pulmonary Disease | 24 | 119 | **20%** |
| Radiation Oncology | 0 | 1 | **0%** |
| Rheumatology | 18 | 148 | **12%** |
| Sports Medicine | 0 | 11 | **0%** |
| Thoracic Surgery | 0 | 2 | **0%** |
| Urology | 15 | 127 | **12%** |
| Vascular Surgery | 2 | 13 | **15%** |
| **Podiatry Providers** | **61** | **316** | **19%** |
| **Uncategorized Providers** | **169** | **1307** | **13%** |
| **Total** | **2879** | **21093** | **14%** |
